# Automating advocacy: creating local alcohol harm risk profiles to assist community input into alcohol license applications

**DOI:** 10.1080/03036758.2024.2412516

**Published:** 2024-12-12

**Authors:** Jessie Colbert, Nick Young, Daniel J. Exeter

**Affiliations:** a School of Population Health, University of Auckland, Auckland, New Zealand; b Centre for eResearch, University of Auckland, Auckland, New Zealand

**Keywords:** Deprivation, community advocacy, alcohol-related harm, IMD18, NZDep, geography, alcohol licensing, data zones, inequities

## Abstract

The physical (spatial) and temporal availability of alcohol is a key determinant of alcohol use and harm. Community input into local alcohol licensing decisions is vital and may be supported by online tools that provide information on alcohol harm risk for local areas. We developed an online tool to provide data on area-level factors (deprivation and ethnic composition) shown to increase a community's risk of alcohol-related harm from the surrounding density and proximity of licensed premises. Data were derived using the Index of Multiple Deprivation (IMD18), the New Zealand Index of Deprivation (NZDep18), and the New Zealand Census 2018. In addition, we mapped proximity to sensitive sites (schools, hospitals and Marae [Māori meeting grounds]) and existing licensed alcohol premises. In this paper, we demonstrate the development and use of our automated alcohol reporting tool that integrates numerous secondary data sources related to alchohol-related risks in the community within a 1 and 2 km radius (buffer) of an address seeking an alcohol off-license. Online tools leveraging geospatial data may assist community-based organisations to actively participate in local alcohol licensing decision processes using a robust evidence-base and support efforts to ensure that the impact of licensing decisions on equity is explicitly considered.

## Introduction

The physical (spatial) and temporal availability of alcohol is a key determinant of alcohol use and harm. The greater the number of licensed premises in a community and the longer their trading hours, the greater the risk the community will experience alcohol-related harm (Popova et al. 2009). New Zealand research shows that the number and density (per capita or per kilometre road network) of licensed premises are positively associated with a range of alcohol-related harms, including violence (Cameron et al. 2017), motor vehicle accidents (Cameron et al. 2012a), and heavy adolescent drinking (Huckle et al. 2008). Alcohol sold late at night is also more likely to be purchased by heavier and more frequent drinkers (Casswell et al. 2014).

Local communities shoulder the majority of alcohol-related harms from weak regulation of the physical and temporal availability of alcohol, in part due to the inequitable distribution of off-license outlets (i.e. takeaway alcohol sales) (Hay et al. 2009) and living in closer proximity to alcohol outlets (Hobbs et al. 2020). Increased alcohol availability encourages competition between retailers resulting in lower alcohol prices and longer trading hours (Cameron et al. 2012b). Increased visibility of licensed premises may also promote norms around alcohol consumption in a community (Kuntsche et al. 2008). Further, advertising at alcohol retail outlets increases the harmful exposure to alcohol advertising (Kuo et al. 2003; Pasch et al. 2007) and contributes to degradation of neighbourhood environments (New Zealand Law Commission 2010).

Disproportionate exposure to alcohol risk environments can perpetuate longstanding alcohol harm inequities in New Zealand, particularly experienced by Māori (Indigenous people of New Zealand) and Pacific communities. Almost one in every five New Zealanders aged 15 years and over were classified as hazardous drinkers in 2021/22 (Ministry of Health 2022), but this prevalence was one in three among Māori. In 2007, Māori had an age-standardised premature death rate from alcohol more than twice that of non-Māori (Connor et al. 2015), with higher proportions of cancer among Māori than non-Māori found to be attributable to alcohol use (Connor et al. 2017).

Spatial variations in the relationship between the availability of alcohol and harm are well established. Characteristics of local communities that moderate the association between outlet density and harm include area-level deprivation (Gruenewald et al. 2006; Livingston 2008; Pridemore and Grubesic 2012a; Karriker-Jaffe et al. 2018), population density (Cameron et al. 2013), age structures (Ayuka et al. 2014), presence of social housing and/or vacant land use nearby (Pridemore and Grubesic 2012b), and ethnic composition (Ayuka et al. 2014). Other groups vulnerable to alcohol outlet density and proximity include children, young people and persons with alcohol use disorders. Many jurisdictions around the world implement evidence-based alcohol-license restrictions (e.g. Huckle et al. 2008; Chen et al. 2010; Rowland et al. 2014), such as proximity controls, prohibiting new alcohol outlets opening in close proximity to sensitive sites such as schools, churches and hospitals, or to limit the potential impact of license density on treatment-related outcomes and recovery for persons with alcohol use disorders (Stahler et al. 2007).

Reducing the physical and temporal availability of alcohol is one of the most effective and cost-effective measures to reduce alcohol consumption and harm, alongside increasing alcohol prices and restricting alcohol marketing (Babor et al. 2022). The Sale and Supply of Alcohol Act (2012), facilitated the development of Local Alcohol Policies (LAPs) and consequently devolved decision-making on alcohol license applications to Territorial Authorities (i.e. local government) via District Licensing Committees; while enabling local community members to object to new licensed premises opening, and contesting the granting of license renewal of existing premises. However, The Sale and Supply of Alcohol Act (2012) purpose is to ensure: (i) the sale, supply and consumption of alcohol should be undertaken safely and responsibly and (ii) the harm caused by excessive or inappropriate consumption of alcohol should be minimised. Despite this, no off-license applications were declined during 2019/20 by the District Licensing Committee in New Zealand's largest city, Auckland (Auckland Council 2021). At the time of writing (2023), there are over 11,000 businesses selling alcohol in New Zealand, including a large number of on-licenses (pubs, bars, nightclubs, restaurants), as well as off-licenses (bottle stores, supermarkets, online retailers, etc.) and club licenses (sports clubs, Returned Services Associations, etc.) (Alcohol Regulatory and Licensing Authority 2021). In 2023, an amendment to The Sale and Supply of Alcohol Act (2012), specifically section 203A(2)(d) of the Sale and Supply of Alcohol (Community Participation) Amendment Act 2023, has paved the way for greater community participation in Alcohol licensing decisions, and increased the powers available to Māori to incorporate tikanga Māori into the District Licensing Committee proceedings (Maynard 2024). In light of the recent changes in New Zealand licensing law, there is a need for easy-to-use information portals and tools to support communities who are engaging with the alcohol licensing process.

Communities face numerous difficulties when preparing an objection to an alcohol license. Enabling community engagement in local alcohol licensing decisions is essential but requires the adequate provision of appropriate support and resourcing in the form of guidance, skill development, funding and time (Reynolds et al. 2020). Reynolds et al. (2020) also suggest that presenting a range of evidence is beneficial, in which data can support the storytelling of personal experiences of alcohol harm that is particularly powerful in licensing hearings. In New Zealand, most of the statistics and other data the community requires to build their case to object against a proposed license is available online. For example, New Zealand Census data on population count and ethnicity is provided by Stats NZ (Stats NZ 2024a), New Zealand Deprivation Index and alcohol hospitalisations data is available through Environmental Health Intelligence New Zealand (EHINZ) (Massey University 2024), and Alcohol availability data is provided by Alcohol Regulatory and Licensing Authority (ARLA) (Ministry of Justice 2017). However, the specialised nature of each piece of data required to access, analyse and interpret in a timely manner may be challenging, particularly for community advocates with little or no relevant experience. Indeed, communities can readily describe the challenges their peers are experiencing, but the disparate nature of the supporting evidence may impede their case.

The importance of local data for alcohol licensing decision-making has catalysed the development of geospatial tools that inform assessments of the risk of harm to the local community. Maps can be used to identify area-level deprivation, proximity to relevant amenities, and the location of existing licensed premises to understand the cumulative impact of additional sites (Reynolds et al. 2018). Online spatial data visualisation tools have been considered an acceptable method of communicating effectively with a range of audiences, especially non-experts, for their ability to aid the importance of story-telling (Armstrong-Moore et al. 2021). Developer credibility, use of high-quality research and robust data, and use and endorsement by external bodies are strong determinants of the acceptability of tools by decision-makers (Armstrong-Moore et al. 2021). Tools must also stand up from a legal perspective (Armstrong-Moore et al. 2021), especially as local decisions can be appealed to higher authorities such as ARLA in New Zealand.

An existing spatial tool for alcohol harm in New Zealand, known as EHINZ (Massey University 2024), provides data on local alcohol outlet density, deprivation, hospital admissions and mortality wholly attributable to alcohol, heavy episodic drinking prevalence and hazardous drinking prevalence. These data are presented at the aggregate level, for ‘neighbourhoods’ represented as ‘Census Area Units’ (CAUs, n = 2,004, mean population = approximately 2,200 people in 2013, (Stats NZ 2018, Stats NZ 2024b)) for alcohol outlet density, and local government geographies such as Territorial Authorities (n = 67, mean population = 70,145, excluding Area Outside Territorial Authority and Local Board Areas (Stats NZ 2024c)) and Territorial Authorities and Local Board Areas (TALBs, n = 88, mean population = 71,266, excluding Area Outside Territorial Authority (Stats NZ 2024c)), and health geographies such as District Health Boards (n = 20, mean population = 234,979, excluding Area Outside District Health Board (Stats NZ 2024c)). However, the EHINZ website does not allow users to enter the address of the premises or build customised reports.

This paper describes the development of an automated tool that generates a report centred on the locality of the premises in question and its surrounding neighbourhood, which has the potential to assist community members in their objections to alcohol license applications. The tool generates local data on two moderators of the effects of outlet density on local alcohol harm: deprivation and ethnic composition. The alcohol reporting tool can be accessed by community objectors and/or regulatory agencies at https://imdmap.auckland.ac.nz/.

## Materials and methods

### Deprivation measures

There are two commonly used measures of area deprivation in New Zealand, the New Zealand Index of Multiple Deprivation (IMD) and the New Zealand Deprivation Index (NZDep). The 2018 IMD (IMD18) is a set of tools for identifying concentrations of deprivation in New Zealand. The IMD18 comprises 29 indicators grouped into seven domains of deprivation: Employment, Income, Crime, Housing, Health, Education and Access to services. The IMD18 is the combination of these seven domains, which may be used individually or combined. The IMD18 measures deprivation among 6,181 small areas known as data zones, designed to be intermediary zones between Statistics New Zealand's small area geographic scales Statistical Area 1 (SA1) and Statistical Area 2 (SA2).

The 2018 NZDep (NZDep18) is another measure of deprivation available in New Zealand, derived from the 2018 census data (Atkinson et al. 2020). The NZDep18 comprises nine indicators representing eight dimensions of deprivation (communication, income, employment, qualifications, home ownership, support, living space, and living conditions). There are two important differences between the IMD18 and the NZDep18. First, the majority of indicators for the IMD18 are derived from routine administrative data, while the NZDep18 is based solely on census data. This means that for the NZDep18, there are some locations in New Zealand that do not have a value reported due to suppressed population data. Second, the smallest geographic boundaries used to report the NZDep18 (SA1s) are smaller than data zones. Therefore, in the Alcohol Harm Risk Profile tool, herein referred to as the risk-reduction tool, a population-weighted version of the NZDep18 calculated at the data zone level is used. As we needed a deprivation value to be reported for any input address in New Zealand in the tool and output report, we decided to use the IMD as the primary measure of deprivation used in the tool. However, given the IMD is a relatively new measure of deprivation in New Zealand, we also included a section in the risk-reduction tool output report that provides the reader with the decile of the NZDep18. The NZDep18 is published at two geographic scales: Statistical Area 1 (average population approximately 100 to 200) and Statistical Area 2 (average population approximately 2,000), approximately representing a few houses or streets and suburbs, respectively. We report the NZDep18 at the Statistical Area 1 scale.

## Data

Data sources used in the tool and output report are outlined below in Table 1.

**Table 1.** Data sources used in the tool and output report.

Data source	Description	Usage	Source
Schools	New Zealand Schools dataset, 2022, from Ministry of Education	Sensitive site	Ministry of Education, 2022
Hospitals	New Zealand hospital locations, 2020	Sensitive site	Marek et al., 2020
Marae	Marae locations, 2022 refresh from Te Kāhui Māngai	Sensitive site	Te Puni Kōkiri, 2022
Licensed alcohol premises	GeoHealth laboratory dataset sourced from the Alcohol Regulatory and Licensing Authority (ARLA) for current and active licensed premises for the period of 2015 to 2018	licensed alcohol premises	Deng et al., 2020
New Zealand Index of Multiple Deprivation 2018 (IMD18)	Neighbourhood-level deprivation index	Area deprivation measure, primary	Exeter et al., 2017
New Zealand Deprivation Index 2018 (NZDep18)	Area-level deprivation index	Area deprivation measure, secondary	Atkinson et al., 2020
New Zealand Census data 2018	Total response ethnicity for Māori, Pacific Peoples and other ethnicities	Ethnic composition	Stats NZ, 2020 (accessed through the Integrated Data Infrastructure)

### Tool description

**Figure 1.**
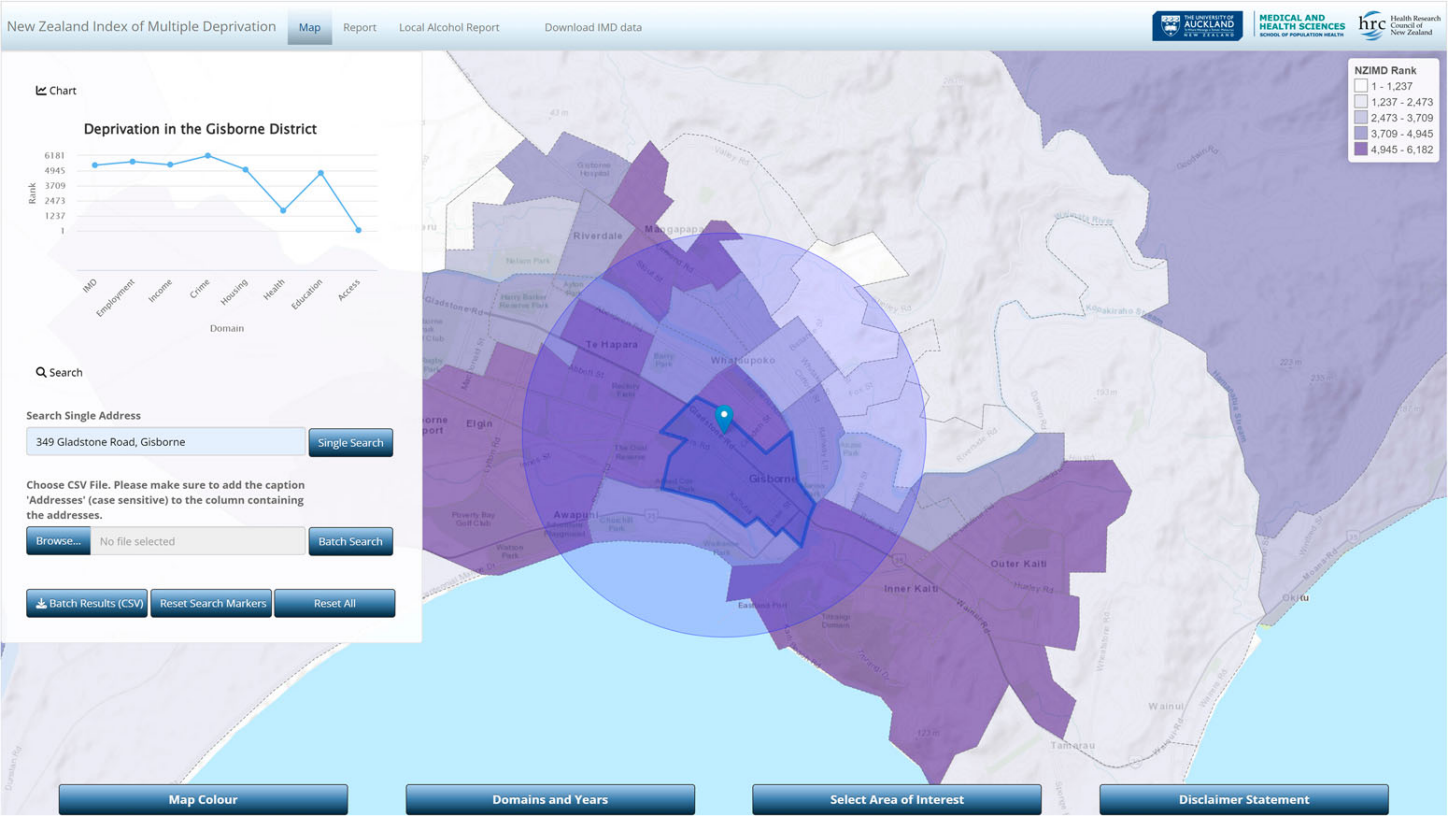
IMD18 map and deprivation chart displayed when an input address is entered into the ‘Search Single Address’ function in the R Shiny application web interface. Example for the location of 349 Gladstone Road, Gisborne.

When a user navigates to https://imdmap.auckland.ac.nz/ in their browser, they are presented with an advanced interactive IMD18 map. The search tool on the left-hand side (Fig. 1) allows users to search for a street address. When a user enters an address, the address is geocoded (converted from street address to latitude and longitude), and a marker is displayed at that position on the map. Additionally, a circle of radius 2 km is shown centred on that marker. Increasingly, a 2 km buffer (circle) around a proposed off-license site is being used to define the ‘locality’ by District Licensing Committees, so we indicate this area on the map. The data zones intersecting that circle are used for surrounding context in later report generation. A deprivation chart of the IMD18 and its seven domains for the data zone where the input address is located is displayed in a pane on the left-hand side of the web interface. The IMD ranks areas in ascending levels of deprivation from 1 to 6,181. Typically, ranks of deprivation are often categorised into deciles (1 to 10) or quintiles (1 to 5), with the higher values representing increasing levels of deprivation. Quintiles can convey patterns on a map more quickly than deciles. Therefore to ease interpretation, we divided the IMD ranks into quintiles, with Quintile 5 representing the most deprived 20% of data zones in New Zealand (IMD ranks 4,945 to 6,181).

If the user then clicks on the ‘Local Alcohol Report’ tab, they are presented with a field to enter their email address. After clicking ‘send’, a PDF report is dynamically generated and emailed to the user, and the user can return to the https://imdmap.auckland.ac.nz/ home page. Here, we demonstrate the process for 349 Gladstone Road, Gisborne.

The generated report describes the alcohol harm risk profile of the area surrounding the entered address. It includes a map showing the address and surrounding data zones with locations of interest plotted (licensed premises, schools, hospitals and Marae, if relevant), a plot showing the seven indices of multiple deprivations for the data zone containing the entered address, and a table comparing the data zone with the rest of New Zealand (see supplementary material). The map is dynamic, so the presence of locations of interest is included if relevant (within the area of interest). Our intention is for the automated report to be a stand-alone document that could supplement a brief of evidence submitted by the community objector or organisation. The report therefore provides a summary table of the deprivation ranks and a brief description for the IMD and its domains.

The report presents the highest and lowest deprivation scores of any data zone located within 1 and 2 km of the address to align with the decision process, which typically requires the level of harm in the locality (usually 1 to 2 km) of the proposed premises to be examined. This interpretation of the ‘locality’ (as it relates to section 105 of the Sale and Supply of Alcohol Act (2012)) contrasts the evidence demonstrating that the harms from alcohol availability extend well beyond this distance (Kypri et al. 2008). The proximity of the entered address to sensitive sites and licensed alcohol premises in the surrounding area is presented, including the count of locations, and the minimum, average (mean) and maximum distance from the entered address to sites or premises located within 1 km or 2 km. Sensitive sites considered are schools (Ministry of Education 2022), hospitals (Marek et al. 2020) and Marae (Te Puni Kōkiri, 2022). Marae are significant sites in indigenous Māori culture, utilised as sacred gathering places (Clark et al. 2014) and communal complexes used for everyday Māori life (King et al. 2015). Proximity information for licensed alcohol premises is broken down by license type. Licensed alcohol premises data were sourced from the GeoHealth laboratory (Deng et al. 2020), which were sourced from ARLA for current and active licensed premises for the period of 2015 to 2018.

The ethnic composition of the data zone comprising the entered address is described in the output report, using data from the New Zealand Census 2018 to calculate the percentage of Māori, Pacific Peoples and Other ethnicities in the data zone, and compare these percentages to the equivalent ethnic percentages for the total New Zealand population. We used the ‘Total Response’ ethnicity coding for this analysis. Therefore, an individual who identified themselves in the Census as Māori and Samoan would be classified as being in both the ‘Māori’ and ‘Pacific Peoples’ categories. The total response output ensures all ethnic groups that an individual report is counted, however, this also results in the overall total of the three ethnic groups combined potentially being greater than 100%.

In this paper, we use the address of an application for a proposed off-license outlet in a deprived area of a small city in New Zealand, 349 Gladstone Road, Gisborne, to demonstrate the components of the report. A full copy of the report is available as supplementary material.

### Tool development

The web interface for the alcohol harm risk profile is an R Shiny application. Geocoding is handled by the geocode function from the ggmap package, using the Google Maps API. The report is based on a template written in R Markdown (Rmd). This Rmd file expects several parameters with information specific to a given location. R code chunks within the Rmd file are executed by knit function provided by the knitr package, which is in turn called by the render function from the rmarkdown package. The render function then calls pandoc to convert the generated Markdown (md) file into an intermediary LaTeX (tex) file. The Rmd file also uses several LaTeX directives. This tex file is then converted to PDF by pandoc. The generated PDF is then emailed as an attachment with the send.mail function from the mailR package. The packages used are as listed in Table 2.

**Table 2.** Packages used in the development of the R Shiny application.

Package name	Version	Description	Purpose
shiny	1.7.4	Web Application Framework for R	IMD map website
ggmap	3.0.0.9001	Spatial Visualization with ggplot2	Geocoding
rmarkdown	2.22	Dynamic Documents for R	Rendering report PDF
knitr	1.43	A General-Purpose Package for Dynamic Report Generation in R	Executing code chunks in Rmd file
lubridate	1.9.2	Dates and times made easy	Extracting retrieval date of report
sp	1.6-1	Classes and Methods for Spatial Data	Loading spatial data
kableExtra	1.3.4	Construct Complex Table with ‘kable’ and Pipe Syntax	Tables in the report
readxl	1.4.2	Read Excel files	Read NZDep data
Orcs	1.2.3	Omnidirectional R Code Snippets	Converting coordinates to SpatialPolygons with the coords2Polygons function
RColorBrewer	1.1-3	ColorBrewer Palettes	Colours for maps in the report
tmap	3.3-3	Thematic Maps	Rendering out maps in the report
ggplot2	3.4.2.9000	Create Elegant Data Visualisations Using the Grammar of Graphics	Rendering out line graph plots in the report
dplyr	1.1.2	A Grammar of Data Manipulation	Data filtering and processing
mailR	0.8	A Utility to Send Emails from R	Sending the report to the user via email

A PDF of the generated report referred to in-text for address 349 Gladstone Road, Gisborne, is attached as a separate supplementary material file.

Given that the tool takes any input address anywhere in New Zealand, we added a handler for areas where there are no sensitive sites or licensed alcohol premises within 2 km of the geocoded input address. In this scenario, the report and figures within the report will still be generated, but with no data points plotted on the map, and NA values are reported for the distances in the table of proximity to sensitive sites and licensed alcohol premises.

When developing the tool and output report, we took into consideration the user experience and interface when using the tool and the generated report. We aimed to make the tool and report easy to use and intuitive, while also being specific and including detailed information suitable for the overall purpose of the work.

## Results

We used the address of an application for a proposed off-license outlet in a deprived area of a small city in New Zealand to demonstrate the output from the local risk-reduction tool. The generated report output by the tool (https://imdmap.auckland.ac.nz/, Local Alcohol Report tab) describes the alcohol harm risk profile of the data zone in which the address is located and those data zones within 1 and 2 km of the entered address.

**Figure 2.**
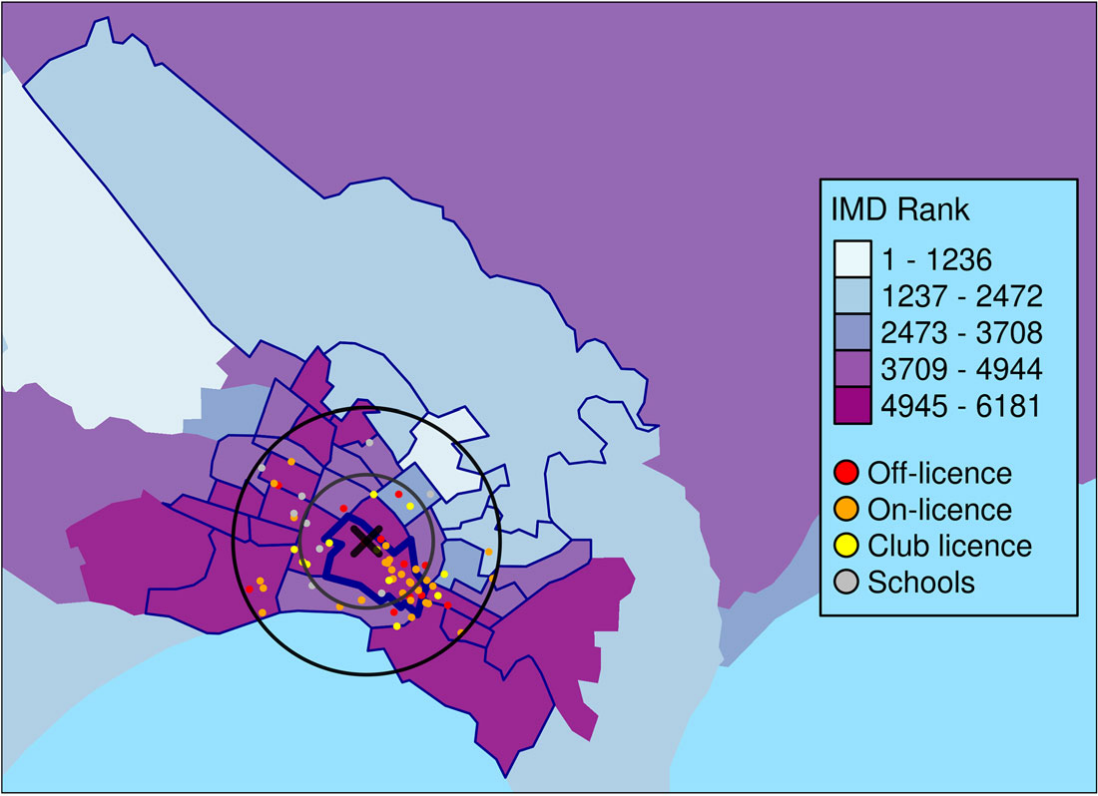
The area deprivation characteristics for the data zone in which 349 Gladstone Road, Gisborne (‘X’), is located and the surrounding neighbourhoods, including 1 and 2 km buffers. Excerpt from the generated report for the location of 349 Gladstone Road, Gisborne.

The first section of the report introduces the IMD and provides an overview of the deprivation profile for the data zone in which the address of interest is located. Figure 2 provides a map of overall deprivation (IMD) for the neighbourhood surrounding the selected address, and locations of interest, if relevant. At the time of writing, there are no hospitals or Marae within 2 km of the proposed premises, so only points for schools and licensed alcohol premises are shown on the map.

**Figure 3.**
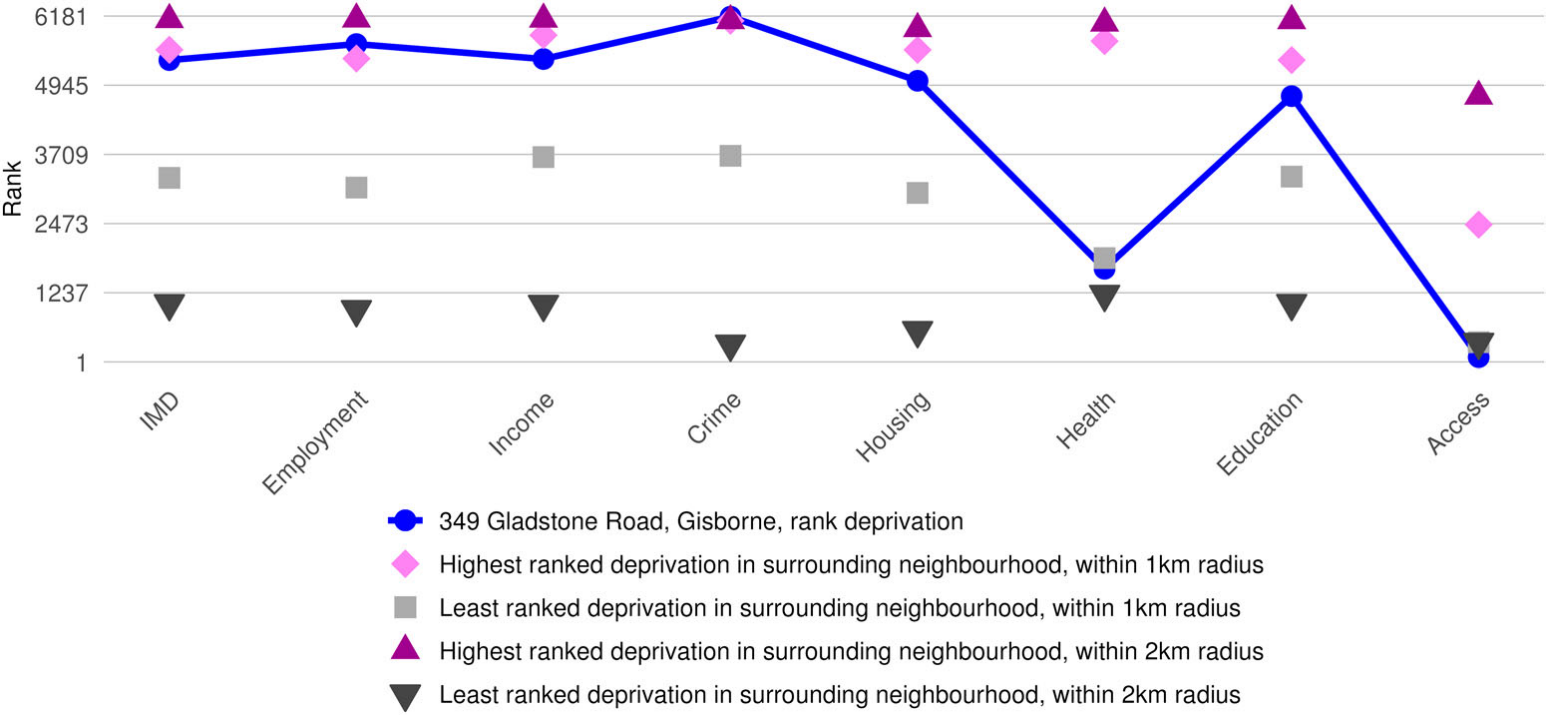
Example plot of the rankings of the IMD and its seven domains for the data zone of interest (solid line) and as well as the highest and least deprivation rankings within 1 and 2 km of the address entered. Excerpt from the generated report for the location of 349 Gladstone Road, Gisborne.

Figure 3 shows that the proposed premises is ranked 5,394 out of 6,181 neighbourhoods in New Zealand according to the IMD. The IMD rank for our area of interest indicates that this particular data zone is in decile 9, which as seen in Figure 2, indicates that the area is in quintile 5. Moreover, Figure 3 shows that while the data zone in which 349 Gladstone Road, Gisborne, is located has relatively high levels of deprivation for most domains, Health and Access are much less deprived. The report also provides the ranks of the IMD and each domain, and describes its level of deprivation in relation to the circumstances within 1 and 2 km of the location of interest (Figure 3). For example, while the data zone of interest is among the highest 0.2 per cent of neighbourhoods in New Zealand with a rank of 6,170/6,181 in terms of Crime deprivation, it is among the least deprived (1.4%) in relation to access to community amenities.

To inform an understanding of the risk within 1 and 2 km from the address, the report also provides the deprivation ranks for the highest and lowest ranked IMD, and its seven domains, for any data zone in the 1 and 2 km area of interest. Interestingly, the area surrounding the data zone in which 349 Gladstone Road, Gisborne, is located also has high levels of employment, income, crime, housing and education deprivation. At the level of a 2 km buffer, there is much greater variability across the deprivation domains.

**Figure 4.**
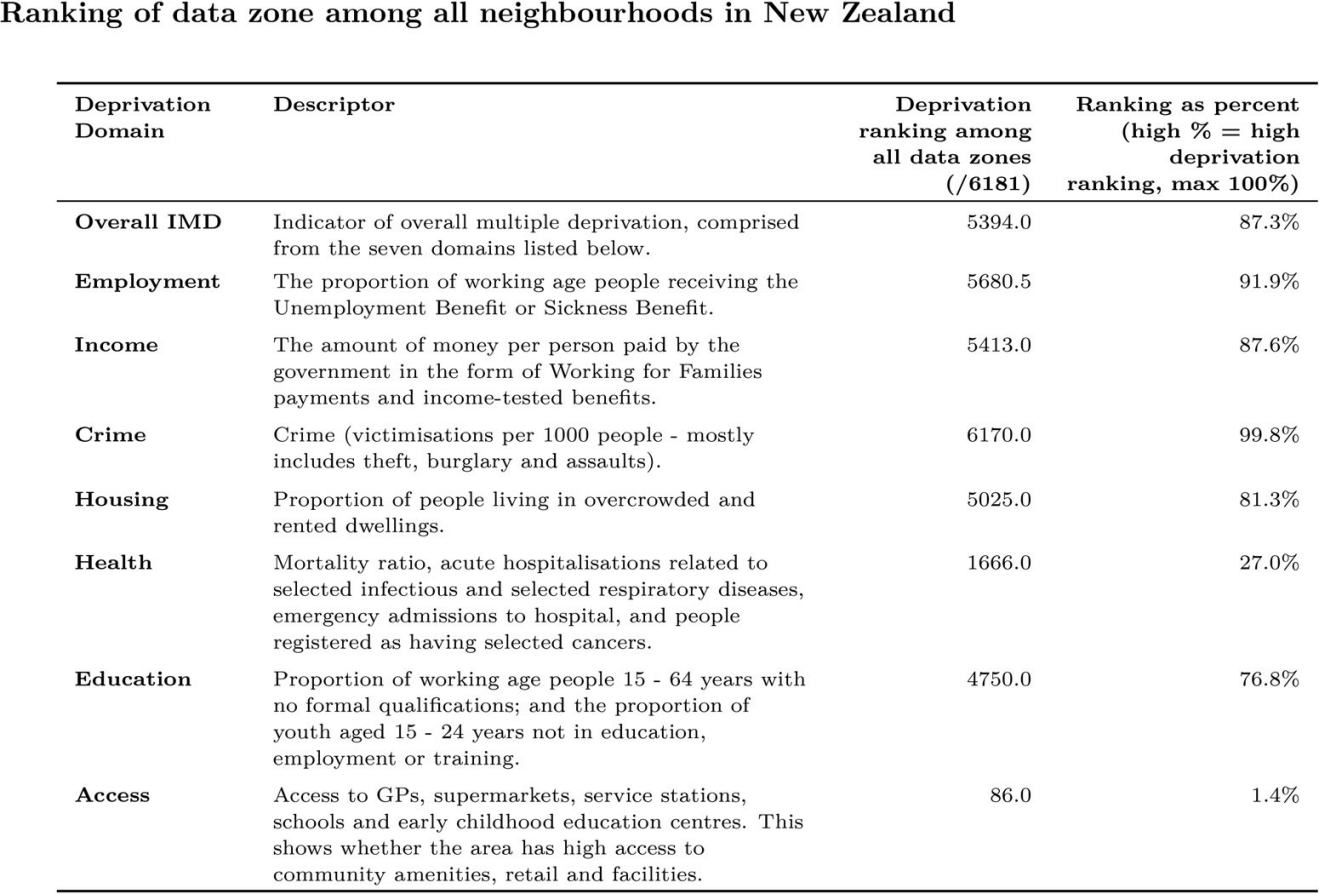
Table showing the deprivation domains for the data zone in which our premises address is located. Excerpt from the generated report for the location 349 Gladstone Road, Gisborne.

The summary table of the deprivation ranks for the IMD18 domains for the input address is shown in Figure 4, comparing the ranking for the premises address to all data zones in New Zealand. Figure 4 shows, for example, that our premises address is in the 87th IMD percentile for overall deprivation, but is much worse (more deprived) for Employment (91.9%) and Crime (99.8%) and considerably lower (less deprived) in terms of Education (76.8%), Health (27.0%) and Access (1.4%).

The NZDep18 is reported for the input address. The NZDep18 decile for the Statistical Area 1 in which 349 Gladstone Road, Gisborne, is located is 10 (the most deprived deprivation decile in New Zealand), while the Statistical Area 2 is decile 9. This is comparable to the IMD18 which, at the data zone level, also reports decile 9 for the premises address.

**Figure 5.**
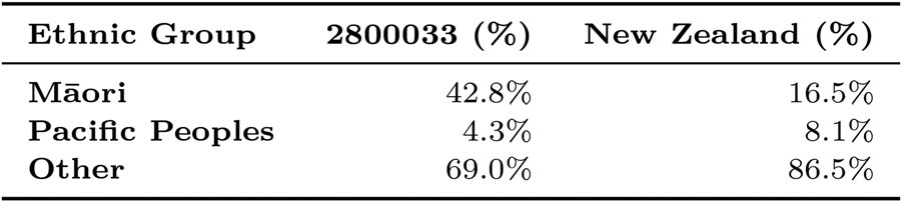
Table showing the ethnic composition of the data zone that 349 Gladstone Road, Gisborne, is located in, using New Zealand Census 2018 data. Excerpt from the generated report for the location of 349 Gladstone Road, Gisborne.

The ethnic composition of the data zone for the input address is described using a table, shown in Figure 5. For example, Figure 5 shows that the population living in the data zone in which 349 Gladstone Road, Gisborne, is located comprised 42.8% Māori, 4.3% Pacific Peoples and 69.0% of people from Other ethnicities. This indicates that the data zone in which 349 Gladstone Road, Gisborne, is located has a different ethnic composition compared to the New Zealand total population, with a higher percentage of residents of Māori ethnicity, and a lower percentage of Pacific Peoples and Other ethnicities.

In addition to location-specific deprivation and ethnic composition characteristics of an entered address, the report links readers to a website of a non-governmental organisation that details the international and national evidence base as it relates to the impact of deprivation and ethnic composition on the relationship between alcohol availability and harm.

**Figure 6.**
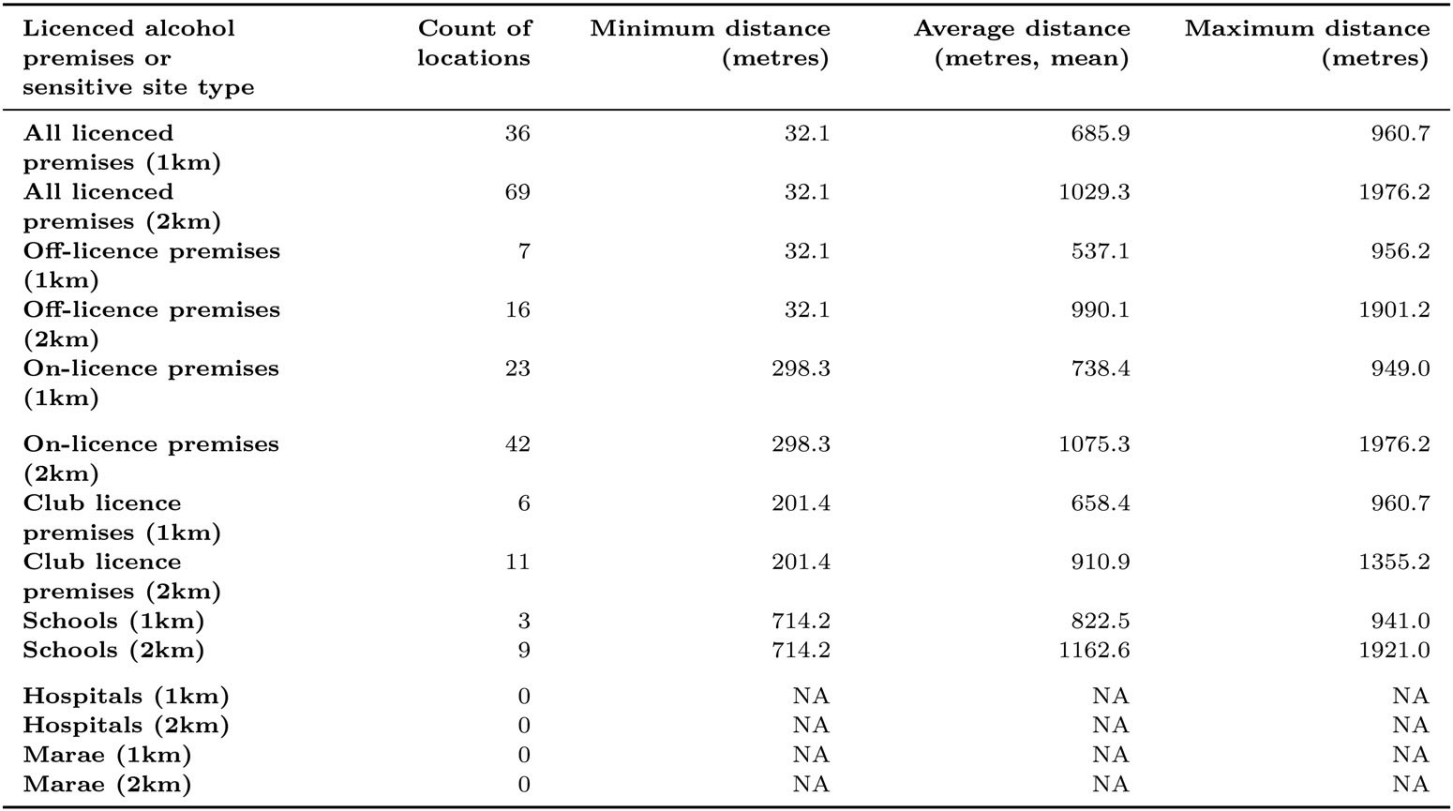
Table showing the proximity of 349 Gladstone Road, Gisborne, to sensitive sites and licensed alcohol premises in the surrounding area. Excerpt from the generated report for the location of 349 Gladstone Road, Gisborne.

The distribution of sensitive sites such as schools, Marae and other licensed alcohol premises is not uniform. Therefore, the report generated is dynamic and presents statistics for these sites when at least one of these amenities falls within 1 km or 2 km of the address of interest. At the time of writing, there were no hospitals or Marae within these proximities of the proposed licensed premises at 349 Gladstone Road, Gisborne, although there were nine schools and over 60 other licensed alcohol premises within 2 km (Figure 6). Figure 6 presents the geographic proximity of these sites to the proposed outlet location, including information on the count of locations, and minimum, average (mean) and maximum distance of the sites to the 349 Gladstone Road, Gisborne, location. The closest licensed premises is only 32.1 metres away from the proposed premises location, while the farthest licensed premises is located 1,976.2 metres away. Despite the large number of licensed alcohol premises within 2 km, the average distance of these sites to 349 Gladstone Road, Gisborne, is over 1 km away (1,029.3 metres). This can be seen in Figure 2, where most of the licensed premises appear to be clustered to the Southeast of the proposed premises, centred around 1 km away from the proposed premises. Schools in the surrounding area have a dispersed geographic distribution (Figure 2), ranging in distance from 714.2 metres to 1,921.0 metres away from 349 Gladstone Road, Gisborne (Figure 6).

The report automatically provides an alcohol-harm risk profile of the area surrounding an entered address. The risk profile generated in the report, tables, figures and supporting information could be used as initial evidence to support objections to new or renewal alcohol license applications in New Zealand. This information and evidence should be used in combination with local information provided by regulatory agencies, the community, the applicant, and the Licensing Committee's own inquiries. The online tool is quick and easy to use, and we hope will be valuable to those who wish to object to a liquor license application within the statutory timeframe.

## Discussion

Despite New Zealand's liquor laws prioritising community input into local alcohol licensing decisions, this has been far from realised. Communities continue to take time away from their responsibilities to object to new outlets opening in their neighbourhood, despite the very low chance of success. For example, a recent report found that between 2018 and 2023, there were 9,490 applications for on-, off-, and club licenses in Auckland, of which 110 (1.1%) went to a District Licensing Committee hearing (National Public Health Service – Northern Region, 2024). Moreover, 84 (76%) of the applications before a District Licensing Committee hearing were granted. Recent changes to key legislation have paved the way for increased community participation in the alcohol licensing process, and the incorporation of tikanga Māori (Maynard, 2024). The automated risk-reduction tool described in this paper is easy-to-use and generates a comprehensive report with local area-specific socioeconomic and demographic information from a reliable source which has the potential to be used as evidence in objections to liquor license applications. We expect that the tool will reduce the amount of time spent by agencies in their reporting on applications as well as for communities in their objections to applications. The report will be updated with the release of new census data as well as updates to the IMD and NZDep deprivation measures. Within the report, we provide a link to the latest evidence base into alcohol-related harms in the community and its determinants, which can also be updated over time, as required.

To prevent new a licensed premises from opening, communities typically have to illustrate the harms directly or indirectly caused by excessive alcohol use in their community. The Sale and Supply of Alcohol Act (2012) outlines the harms as including crime, damage, death, disease, disorderly behaviour, illness, injury or impacts on amenity and good order. However, data on these harms is rarely collected at a level required for local alcohol licensing decisions. Our report provides location-specific data directly relevant to the community of interest to a particular alcohol license application. While overall deprivation statitstics are familiar and commonly used in District Licensing Committee decisions, we contend that with further awareness of the tool by communities and District Licensing Committee chairs alike, our enriched profile of the area, including crime- and health-related deprivation and the number of sensitive sites in close proximity to proposed licensed premises may strengthen the community's objection to new premises. We have endeavoured to provide the technical details and implications of our research in a clear and concise manner, and have sought feedback from community members on previous iterations of the report to meet their needs.

As Governments around the world focus on the value of localism (Martineau et al., 2014) in alcohol licensing decision-making, researchers and practitioners are required to find ways to present local information. Online, on-demand tools that synthesise geospatial data present a unique opportunity to assist communities in the daunting process of objecting to local alcohol license applications. Anecdotal reports suggest that experts such as the New Zealand Police and Medical Officers of Health may not have capacity to provide evidence against every license application. Therefore, they may use the area deprivation characteristics in proximity to the proposed licensed premises to determine whether or not to make a submission in opposition to an alcohol license being granted to an applicant by a District Licensing Committee. The geospatial perspective presented in this paper demonstrates the need to consider the sociodemographic and deprivation circumstances of the surrounding neighbourhoods when making such decisions.

The online tool has many potential uses and applications. In licensing decisions, consideration must be given to the impact of a premises on the amenity and good order of the locality. This includes noise levels, nuisance and vandalism, the number of similar licensed premises (e.g. off-licenses) already in the locality, as well as the nearby land use and its purposes of use. This permits decision-makers to consider the proximity of proposed licensed premises to sensitive sites such as schools, hospitals, Marae, churches, social housing, and alcohol treatment services. As such, future additions to the online tool could include expanding upon the already included sensitive sites and including additional sensitive sites. Additional context, such as noise complaints, graffiti removal, alcohol-related crashes, ambulance response patterns for alcohol-related calls, and alcohol-related Emergency Department presentations could also be integrated in future updates.

While this paper provides opportunities to encourage communities to participate in licensing decisions in their locality, this research is not without its limitations. A key limitation is the timeliness and accuracy of the data used in the report. Indeed, this is a fair criticism of nearly all social and population health research that utilises the census for denominator populations. The IMD is readily updateable using the routine datasets within the Integrated Data Infrastructure (IDI) and the longer-term vision is for the IMD to be updated for intercensal periods. The United Kingdom-based IMDs were developed in the early 2000s to address similar concerns about the relevance of sociodemographic and area deprivation data derived from their decennial censuses, and are updated every 2 to 4 years. In the United Kingdom and New Zealand, there is no routine data source that provides nationally representative housing-related data, so updates are still dependent to some extent on the use of Census data for some indicators. The developers of the 2018 IMD used in this report are encouraged by Statistics New Zealand's experimental administrative population census for its potential use as denominators in the future for New Zealand-based IMD updates.

In addition, the source of data on alcohol licenses (Deng et al., 2020) in the local area has not been examined for its validity. Until a high-quality central register of licenses is developed and maintained, it is imperative that the users of the tool undertake their own data collection of local licensed premises and include this in their license objections. In 2010, the New Zealand Law Commission recommended that ARLA enhance the flow of data and information concerning licensing matters (New Zealand Law Commission, 2010). The development of this tool has clearly showcased the on-going large gap in knowledge regarding the location of premises that sell alcohol in New Zealand. For communities to effectively participate in licensing matters, and for accurate licensing decisions, access to this information is of paramount importance.

In the tool, we use the custom-designed data zones to report the IMD18, and further in the output report we also report the NZDep18 at both the Statistical Area 1 (SA1) and Statistical Area 2 (SA2) scale. Previous research has shown strong correlations between NZDep and the IMD (*R*^2 ^= 0.90, *p*-value < 0.0001) (Exeter et al., n.d.), and as such, you may expect some areas to have slightly different deprivation scores depending on the measure used. In our output report for the example premises address, the reported IMD decile 9 is comparable to the NZDep18's score at the SA2 (suburban) scale. However, a particular strength of the IMD is its ability to not only report the overall level of deprivation, but also the deprivation circumstances of the 7 domains, as shown in Figure 3. Another advantage of the IMD18 and the custom-designed data zones is that, unlike the NZDep18, there is full coverage of New Zealand with no NULL values. In comparison, due to the NZDep18 being based only on data sourced from the New Zealand Census, there are some locations in New Zealand that do not have a value reported. For the tool and report, we needed a deprivation value to be reported for any input address in New Zealand. Therefore, we used the IMD18 and data zones as the primary deprivation measure and spatial scale reported, however, due to the IMD being a relatively new creation and the NZDep18 still being used widely, we included a short section on the NZDep18.

The geographic base used as neighbourhoods in the creation of the IMD (data zones) is an intermediary scale between Statistics New Zealand's new SA1 and SA2 boundaries. Data zones were designed to be large enough to limit the amount of neighbourhood-level statistics suppressed by Statistics New Zealand based on the confidentiality constraints that users of the IDI must adhere to (Exeter et al., 2017; Exeter et al., n.d.). While SA1s have smaller populations than data zones, they were generally too small to provide any detailed indicator information and being larger than data zones, SA2s are generally too large to represent a homogenous notion of ‘neighbourhood’ or locality (Mills et al., 2022). Moreover, there is substantial evidence that changing either the size or configuration of area boundaries can have a dramatic impact on statistical results (Openshaw & Taylor, 1979; Manley et al., 2006). Previous studies have created population-weighted versions of NZDep to ensure both the IMD and NZDep are measured for the same geographic scale, and found that both indices were almost identical in statistical modelling results. Thus, we would likely expect the population-weighted NZDep deciles to be very similar to that reported for the IMD, if a common geographical unit was used. However, harmonising data between different datasets is complex (Norman et al., 2003), and the IMD developers are currently re-assessing the potential for releasing the IMD at the SA2 level, to be more broadly consistent with other official datasets, and being more directly comparable to NZDep.

The 2023 amendment to The Sale and Supply of Alcohol Act not only has the potential to increase community participation in alcohol licensing decisions, but also has provided greater opportunities for Māori to be involved in the process of alcohol licensing. Under the new act, District Licensing Committees are required to put appropriate procedures in place that let the incoporation of tikanga Māori take place in the proceedings (Maynard, 2024). Tikanga Māori has been increasingly incorporated into other legislation in New Zealand, and can help to further foster community participation. Maynard (2024) identified that currently there is a gap in knowledge of tikanga amongst the District Licensing Committee members. We hope that as this gap in knowledge is addressed, greater emphasis on the values and opinions of the local community will be placed in the alcohol licensing process, and the ease of which community members can object and participate in the process will be fostered and prioritised.

Our alcohol reporting tool was developed in conjunction with advocates from non-governmental agencies who regularly support communities that object to the presence of licensed alcohol outlets in their community. While we contend the output report effectively communicate information relating to alcohol-related harms in the community, we also acknowledge that more work is required to educate the potential users of the tool about its provenance and relevance. We intend to host a hui with potential users including iwi, non-governmental agencies, New Zealand Police, Medical Officers of Health, and District Licensing Chairs to ensure that all groups have the expert knowledge required to use this information in future hearings.

Online spatialisation tools have the potential to assist community members to participate in local alcohol licensing decision processes. Ensuring decisions are informed by local evidence on deprivation and ethnic composition can assist to place equity at the forefront of decision-making, thereby assisting to protect communities that experience disproportionately more harm from alcohol.

## Statistics NZ disclaimer

These results are not official statistics. They have been created for research purposes from the Integrated Data Infrastructure which is carefully managed by Stats NZ. For more information about the IDI please visit https://www.stats.govt.nz/integrated-data/. Access to the data used in this study was provided by Stats NZ under conditions designed to give effect to the security and confidentiality provisions of the Data and Statistics Act (2022). The results presented in this study are the work of the author, not Stats NZ or individual data suppliers.

## Ethics statement

This study was given ethical approval by the Chairperson of the Northern × Regional Ethics Committee on 24 August 2011, with ongoing approval granted by the New Zealand Health and Disability Ethics Committees (Ref: NTX/11/EXP/190).

## Supplementary Material

Supplemental Material
