# Supplementary material for: Automating advocacy: creating local alcohol harm risk profiles to assist community input into alcohol license applications
Source: J R Soc N Z. 2024 Dec 12;55(6):1814–32. doi: 10.1080/03036758.2024.2412516 (PMC12315180; doi:10.1080/03036758.2024.2412516)
Supplement: Supplemental Material [file TNZR_A_2412516_SM4593.pdf]

Alcohol Harm Risk Profile of Area Surrounding:

349 Gladstone Road, Gisborne

This report documents the deprivation and ethnic composition characteristics of the local area surrounding 349 Gladstone Road, Gisborne, and the proximity of 349 Gladstone Road, Gisborne to sensitive sites. The rationale for considerations of deprivation and ethnic composition in local alcohol licensing decisions is available here.

Level of deprivation in the surrounding area

a) Index of Multiple Deprivation (IMD)

The IMD was developed by the University of Auckland[1] and measures deprivation at the neighbourhood level in custom-designed data zones. Data zones are larger in population size than meshblocks (approximately 8 meshblocks per data zone) but smaller than Census area units. In urban settings, they are just a few streets long and a few streets wide.

Using the IMD, 349 Gladstone Road, Gisborne, (marked as 'X' in the figure below) is located in data zone 2800033. This data zone or 'neighbourhood' (boundary shown with dark blue line below) has a Census 2018 population of 585 persons. The average population of all 6181 data zones in New Zealand is 761 (range 399 to 1200).

This neighbourhood ranks **5394 of the 6181 neighbourhoods** in New Zealand for overall multiple deprivation. Multiple deprivation comprises 29 indicators grouped into seven domains of deprivation: Employment, Income, Crime, Housing, Health, Education and Access to services.

The IMD ranking of 5394 (Decile 9) places this neighbourhood in the **most deprived 12.7%** of all 6181 neighbourhoods for multiple deprivation.

The level of deprivation in neighbouring data zones within 1km and 2km of 349 Gladstone Road, Gisborne, is also shown in the figure below (grey circle is 1km radius, black circle is 2km radius - darker colour of data zone, higher deprivation). The location of existing alcohol licenced premises[2] (as of 2018) are shown as red dots for off-licence premises, orange dots for on-licence premises, and yellow dots for club licence premises. The location of schools[3] are shown as grey dots. Note schools includes Early Childhood Education Services, Schools and Tertiary providers. The location of hospitals[4] (if relevant, as of 2017) are shown using green dots. The location of Marae[5] (if relevant, as of 2022) are shown using blue dots.

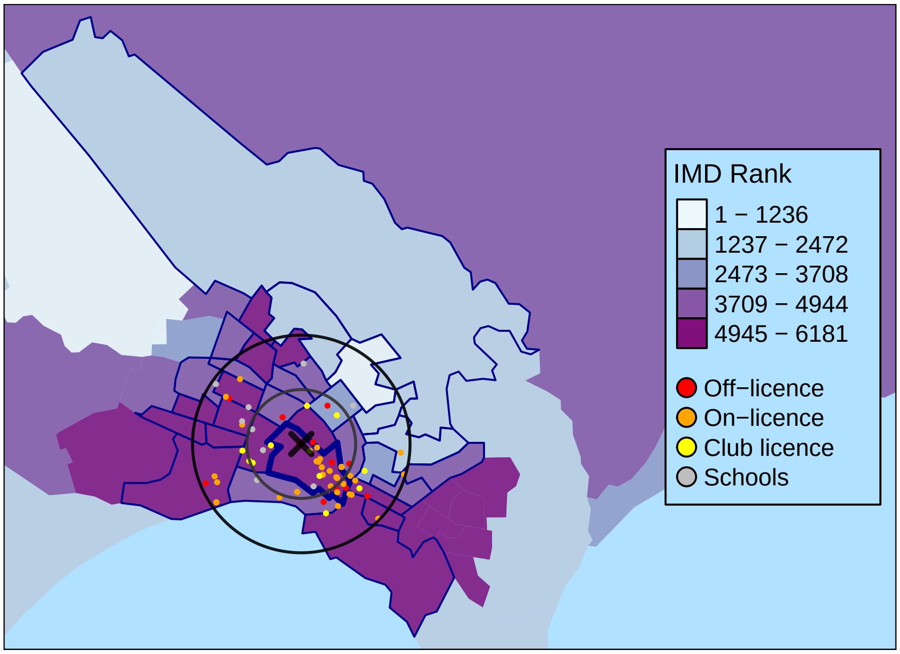

THE UNIVERSITY OF AUCKLAND
Te Whare Wānanga o Tāmaki Makaurau
NEW ZEALAND
MEDICAL AND HEALTH SCIENCES
SCHOOL OF POPULATION HEALTH
hrc Health Research Council of New Zealand

The ranking of each of the seven domains of deprivation in the data zone comprising 349 Gladstone Road, Gisborne, is shown below. This allows for a much clearer understanding of the type of deprivation that is most prevalent in the immediate neighbourhood. The highest and lowest deprivation scores in any data zone within 1km and 2km of 349 Gladstone Road, Gisborne, is also shown. The average (mean) IMD deprivation rank (out of 6181) for those data zones within 1km and 2km of 349 Gladstone Road, Gisborne is 4760.78 and 4534.35, respectively.

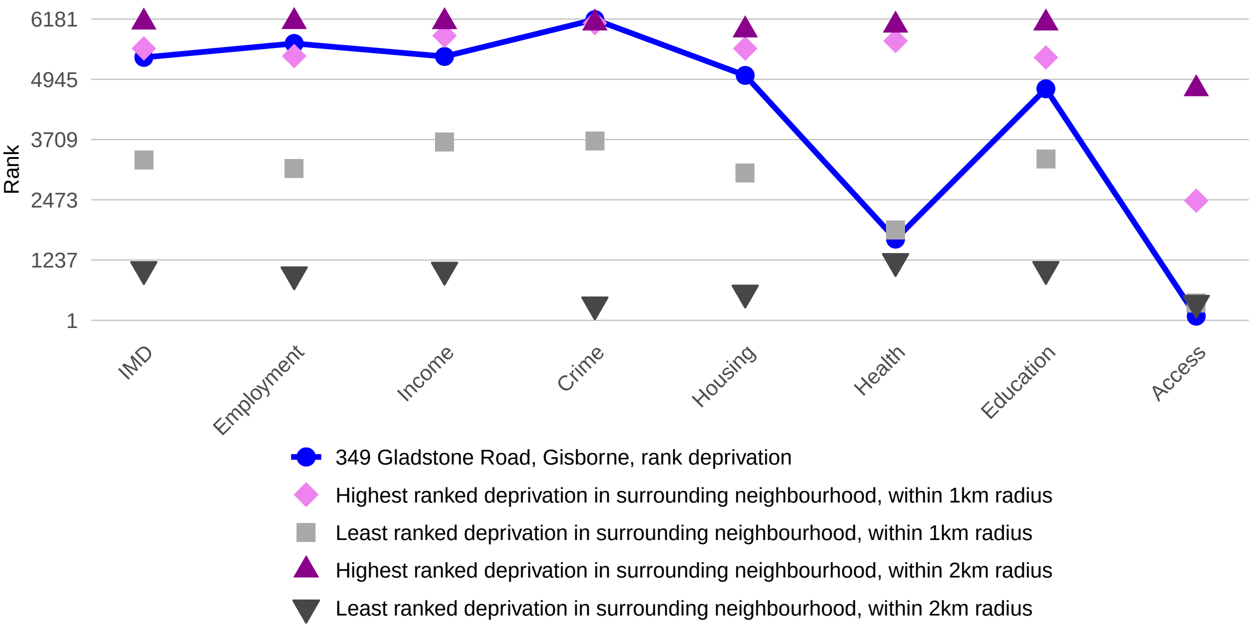

Ranking of data zone among all neighbourhoods in New Zealand

Deprivation Domain	Descriptor	Deprivation ranking among all data zones (/6181)	Ranking as percent (high % = high deprivation ranking, max 100%)
**Overall IMD**	Indicator of overall multiple deprivation, comprised from the seven domains listed below.	5394.0	87.3%
**Employment**	The proportion of working age people receiving the Unemployment Benefit or Sickness Benefit.	5680.5	91.9%
**Income**	The amount of money per person paid by the government in the form of Working for Families payments and income-tested benefits.	5413.0	87.6%
**Crime**	Crime (victimisations per 1000 people - mostly includes theft, burglary and assaults).	6170.0	99.8%
**Housing**	Proportion of people living in overcrowded and rented dwellings.	5025.0	81.3%
**Health**	Mortality ratio, acute hospitalisations related to selected infectious and selected respiratory diseases, emergency admissions to hospital, and people registered as having selected cancers.	1666.0	27.0%
**Education**	Proportion of working age people 15 - 64 years with no formal qualifications; and the proportion of youth aged 15 - 24 years not in education, employment or training.	4750.0	76.8%
**Access**	Access to GPs, supermarkets, service stations, schools and early childhood education centres. This shows whether the area has high access to community amenities, retail and facilities.	86.0	1.4%

b) The New Zealand Index of Deprivation (NZDep)

Another deprivation index developed in New Zealand, by the University of Otago, is the New Zealand Index of Deprivation 2018[6], which comprises nine dimensions of deprivation using data from the Census 2018.

Using the Census geographic boundaries, the Statistical Area 1 (a small area that contains 100-200 people) that comprises 349 Gladstone Road, Gisborne, has a deprivation decile of 10 (decile 1 = least deprived, decile 10 = most deprived).

At the level of Statistical Area 2 (a larger area that equates to a suburb), the deprivation decile is 9.

Ethnic composition in the surrounding area

In the Census 2018, the data zone in which 349 Gladstone Road, Gisborne, is located had the following ethnic composition. Comparison with the New Zealand total population is shown below.

Ethnic Group	2800033 (%)	New Zealand (%)
**Māori**	42.8%	16.5%
**Pacific Peoples**	4.3%	8.1%
**Other**	69.0%	86.5%

The above table includes all people who stated each ethnic group, whether as their only ethnic group or as one of several. Where a person reported more than one ethnic group, they have been counted in each applicable group. As a result, percentages do not add up to 100.

Proximity to sensitive sites and licenced alcohol premises in the surrounding area

The number of sensitive sites and licenced alcohol premises and the minimum, maximum and average distance of these locations using Euclidean distance to 349 Gladstone Road, Gisborne within 1km and 2km is outlined in the table below.

Licenced alcohol premises or sensitive site type	Count of locations	Minimum distance (metres)	Average distance (metres, mean)	Maximum distance (metres)
**All licenced premises (1km)**	36	32.1	685.9	960.7
**All licenced premises (2km)**	69	32.1	1029.3	1976.2
**Off-licence premises (1km)**	7	32.1	537.1	956.2
**Off-licence premises (2km)**	16	32.1	990.1	1901.2
**On-licence premises (1km)**	23	298.3	738.4	949.0
**On-licence premises (2km)**	42	298.3	1075.3	1976.2
**Club licence premises (1km)**	6	201.4	658.4	960.7
**Club licence premises (2km)**	11	201.4	910.9	1355.2
**Schools (1km)**	3	714.2	822.5	941.0
**Schools (2km)**	9	714.2	1162.6	1921.0
**Hospitals (1km)**	0	NA	NA	NA
**Hospitals (2km)**	0	NA	NA	NA
**Marae (1km)**	0	NA	NA	NA
**Marae (2km)**	0	NA	NA	NA

Summary

This report, prepared by the University of Auckland, for the location of the premises (349 Gladstone Road, Gisborne) states that the community in this locality is Decile 9 in deprivation. The community also has a much higher than average Māori population and a lower than average Pacific population. The level of deprivation and ethnic composition of a locality are important indicators of its vulnerability to alcohol-related harm.

References

[1] Exeter DJ, Zhao J, Crengle S, Lee A, Browne M. The New Zealand Indices of Multiple Deprivation (IMD): A new suite of indicators for social and health research in Aotearoa, New Zealand. PloS One 2017; 12: e0181260.

[2] Deng BY, Wiki J, Hobbs M, Marek L, Campbell M, Kingham S. GeoHealth laboratory dataset: cleaned nationwide alcohol outlets 2015–2018. (Version 1) [Data set], 2020. Available from: https://www.canterbury.ac.nz/science/research/geohealth/publications-reports-and-data/

[3] Ministry of Education. New Zealand Schools. [Data set], 2022. Available from (accessed 08/03/2022): https://catalogue.data.govt.nz/dataset/directory-of-educational-institutions/resource/20b7c271-fd5a-4c9e-869b-481a0e2453cd

[4] Marek L, Wiki J, Hobbs M, Campbell M, Kingham S. GeoHealth laboratory dataset: cleaned nationwide hospital locations. (Version 1) [Data set], 2020. Available from: https://www.canterbury.ac.nz/science/research/geohealth/publications-reports-and-data/

[5] Te Puni Kōkiri. Layer_Marae: Marae Shapefile. Update 16th March 2022 (refresh from Te Kāhui Māngai). [Data set], 2022. Available from: https://tpk.maps.arcgis.com/home/item.html?id=3b9e52a2012a4e4cb434e07ce19b36dd

[6] Atkinson J, Salmond C, Crampton P. NZDep18 Index of Deprivation. Wellington; NZ: University of Otago, 2020.

Recommended Citation

Exeter DJ, Colbert J, Young N (2024). Alcohol Harm Risk Profile of Area Surrounding: 349 Gladstone Road, Gisborne. University of Auckland. Retrieved from imdmap.auckland.ac.nz, August 21, 2024.

Further information

For more information about this report contact Professor Daniel Exeter: d.exeter@auckland.ac.nz
